# Glucocorticoids and “Stress” Are Not Synonymous

**DOI:** 10.1093/iob/obz017

**Published:** 2019-07-13

**Authors:** S A MacDougall-Shackleton, F Bonier, L M Romero, I T Moore

**Affiliations:** 1 Department of Psychology, University of Western Ontario, London, Ontario, Canada N6A 5C2; 2 Biology Department, Queen’s University, Kingston, Ontario, Canada K7L 3N6; 3 Department of Biology, Tufts University, Medford, MA 02155, USA; 4 Department of Biological Sciences, Virginia Tech, Blacksburg, VA 24061, USA

## Abstract

Reference to glucocorticoids as “stress hormones” has been growing in prevalence in the literature, including in comparative and environmental endocrinology. Although glucocorticoids are elevated in response to a variety of stressors in vertebrate animals, the primary functions of glucocorticoids are not responding to stressors and they are only one component of complex suite of physiological and behavioral responses to stressors. Thus, the use of the short-hand phrase “stress hormone” can be misleading. Further, simply measuring glucocorticoids is not equivalent to measuring a stress response, nor is manipulating glucocorticoids equivalent to exposing an animal to a stressor. In this commentary we highlight the problems with using functional names for hormones, and of treating cortisol or corticosterone as synonymous with stress. We provide recommendations to add clarity to the presentation of research on this topic, and to avoid conflation of glucocorticoids with stressors and the stress response in the design of experiments.

## Synopsis

Los Glucocorticoides y el “Estrés” no Son Sinónimos (Glucocorticoids and “Stress” Are Not Synonymous) La referencia a los glucocorticoides como “hormonas del estrés” ha aumentado en prevalencia en la literatura, incluso en endocrinología comparativa y ecológica. Aunque los glucocorticoides están elevados en respuesta a una variedad de factores de estrés en animales vertebrados, las funciones primarias de los glucocorticoides no responden a los factores de estrés y son solo un componente de un conjunto complejo de respuestas fisiológicas y de comportamiento a los factores de estrés. Por lo tanto, el uso de la frase abreviada “hormona del estrés” puede ser engañoso. Además, simplemente medir glucocorticoides no es equivalente a medir una respuesta al estrés, ni manipular glucocorticoides equivalente a exponer a un animal a un factor estresante. En este comentario destacamos los problemas con el uso de nombres funcionales para las hormonas y con el tratamiento del cortisol o la corticosterona como sinónimo de estrés. Brindamos recomendaciones para agregar claridad a la presentación de investigaciones sobre este tema y para evitar la combinación de glucocorticoides con factores estresantes y la respuesta al estrés en el diseño de los experimentos. Translated to Spanish by J. Heras (herasj01@gmail.com)

Glucocorticoide und “Stress” Sind Keine Synonyme (Glucocorticoids and “Stress” Are Not Synonymous) Die Bezugnahme auf Glucocorticoide als “Stresshormone” hat in der Literatur zugenommen, auch in der vergleichenden und ökologischen Endokrinologie. Obwohl Glucocorticoide als Reaktion auf eine Vielzahl von Stressoren bei Wirbeltieren erhöht sind, antworten die primären Funktionen von Glucocorticoiden nicht auf Stressoren und sind nur eine Komponente einer komplexen Reihe von physiologischen und Verhaltensreaktionen auf Stressoren. Daher kann die Verwendung der Kurzformel “Stresshormon” irreführend sein. Darüber hinaus ist das einfache Messen von Glucocorticoiden nicht gleichbedeutend mit dem Messen einer Stressreaktion und das Manipulieren von Glucocorticoiden nicht gleichbedeutend damit, ein Tier einem Stressor auszusetzen. In diesem Kommentar werden wir die Probleme bei der Verwendung funktioneller Bezeichnungen für Hormone und bei der Betrachtung von Cortisol oder Corticosteron als Synonym für Stress hervorheben. Wir geben Empfehlungen, um die Präsentation der Forschungsergebnisse zu diesem Thema klarer zu gestalten und die Verschmelzung von Glucocorticoiden mit Stressoren und der Stressreaktion bei der Versuchsplanung zu vermeiden. Translated to German by F. Klimm (frederike.klimm@biologie.uni-freiburg.de)

Glucocorticóides e “Stress” Não São Sinônimos (Glucocorticoids and “Stress” Are Not Synonymous) A referência aos glicocorticóides como “hormônios do estresse” vem se tornando prevalente em literatura, inclusive na endocrinologia comparada e ecológica. Embora os glicocorticóides sejam elevados em resposta a uma variedade de estressores em vertebrados, as funções primárias de glicocorticóides são a de não responder aos causadores do stress e eles são apenas um componente dentro de um complexo conjunto de respostas fisiológicas e comportamentais aos estressores. Assim, o uso da frase curta “hormônio do estresse” pode ser errôneo. Além disso, simplesmente medir glicocorticóides não é equivalente a medir uma resposta ao estresse, nem a manipulação de glicocorticoides é equivalente a expor um animal a um estressor. Neste comentário, destacamos os problemas com o uso de nomes funcionais para hormônios e o tratamento de cortisol ou corticosterona como sinônimos de estresse. Nós provemos recomendações para adicionar clareza à apresentação de pesquisas deste tópico e para evitar a o tratamento direto de glicocorticoides como estressores e a resposta ao estresse no planejamento de experimentos. Translated to Portuguese by Diego Vaz (dbistonvaz@vims.edu)

## Introduction

This commentary seeks to address a problem that we have observed as increasing in prevalence, in our roles as editors, manuscript reviewers, and conference attendees: that of treating the concept of stress as equivalent to glucocorticoid levels. Although we hope the fundamental error of equating stress with hormone levels is rare, the use of the abbreviation “Cort” (referring either to corticosterone or cortisol) as synonymous or interchangeable with “stress” appears to be becoming more common. Although this may seem a superficial, semantic problem of using “short-hand” language to refer to complex biological processes, we argue it is more pernicious. We think this conflation of terms *does* matter, because it affects not only how we describe our studies, but also how we design our experiments, and, perhaps most importantly, how we interpret our results. We thus appeal to investigators to be more specific when describing and presenting their studies so we can all avoid unnecessary confusion and so our field can make stronger advances toward answering important questions.

## The problem with using short-hand

Communication in science requires the deliberate and precise use of language. Often, we use short-hand phrases and jargon when communicating with colleagues in our own subdisciplines, or when communicating with the public to avoid delving into dry complexities. However, when such shorthand is communicated to those not familiar with all of the assumptions, confusion can ensue. This is particularly troublesome when communicating with students and trainees who may then go on to use jargon or shorthand to incorrectly develop new hypotheses in the absence of acknowledging, or even being aware of, important assumptions.

For example, in fields such behavioral ecology, we may make statements such as “male birds sing to defend their territory or attract a mate.” What this statement really means is that there is evidence that over evolutionary time, males that sang more than other males were more successful at excluding conspecific competitors from their territory and attracting mates and thus had greater reproductive success. The shorthand is much more succinct, but could lead one to conclude that natural selection is goal-directed, or that birds are capable of planning for the future.

Functional names are particularly problematic forms of short-hand. For example, some might refer to a gene like FOXP2 as a “language gene” rather than “a gene that encodes a protein whose regulated expression plays a critical role in the development of neural regions that contribute to language development.” The shorthand may be succinct but can lead to the fallacious view that genes directly encode behavioral traits. Similarly, referring to IT15 (huntingtin) as the “gene for Huntington’s Disease” is a shorthand that erroneously implies the gene encodes the disease state. Functional names can also lead us to neglect other causal factors that might be important components of the phenomenon being studied and to ignore other functions of an entity (e.g., a gene) that are not included in the name. For example, regions of the visual and auditory cortex are now known to be involved in multisensory processing (Ghazanfar and Schroeder 2006), which is obscured by functional names that refer to a single sensory modality.

Using functional names for hormones can similarly lead to confusion. In particular, use of the term “stress hormone(s)” to refer to glucocorticoids creates confusion in several important ways. First, the recent practice of referring to glucocorticoids as stress hormones is increasing in prevalence (see below), despite a lack of consensus on what meaning we intend when using this terminology. Second, the term “stress hormone” erroneously implies that the primary function of glucocorticoids is in mediating a stress response and thus ignores and draws attention away from the many other fundamental regulatory functions of these hormones. Further, use of the term might imply that glucocorticoids function to create stress, or are equivalent to stress. Third, elevation of circulating glucocorticoids is only one component of a suite of physiological responses to a stressor, and so referring to glucocorticoids as stress hormones oversimplifies the complex nature of neural and endocrine responses to stressors. Fourth, misuse of the term has led some to equate treating an animal with glucocorticoids with exposing an animal to a stressor. In addition, several researchers who are interested in quantifying stress in their study animals also assume that by measuring glucocorticoids they are measuring stress, leading to a presumption that elevated levels of this hormone are negative, following from the assumption that stress is bad. As editors and reviewers, we have noted an increase in all of these problems in recent years. Below we expand on each of these points. We then further explore how equating glucocorticoids with stress can lead to confusion and potential misinterpretation of results in several ways. We conclude by recommending that we discontinue use of the term “stress” when describing cortisol or corticosterone, and refer more precisely and accurately to the hormones under consideration.

## Use of the term “stress hormones” is increasing

The term “stress hormone” has been prevalent in scientific literature for decades. In early studies it was used to refer to a variety of hormones that change in plasma concentration following exposure to a stressor. For example, the earliest record of “stress hormones” in the Web of Science (Clarivate) core collection (1900–2018) was a publication manipulating adrenalin and hydrocortisone (Rytömaa and Kiviniemi 1969). Other studies used the term stress hormone to refer to glucagon, secretin, or oxytocin (Bloom 1973; Oektedalen et al. 1982; Lang et al. 1983).

**Fig. 1:**
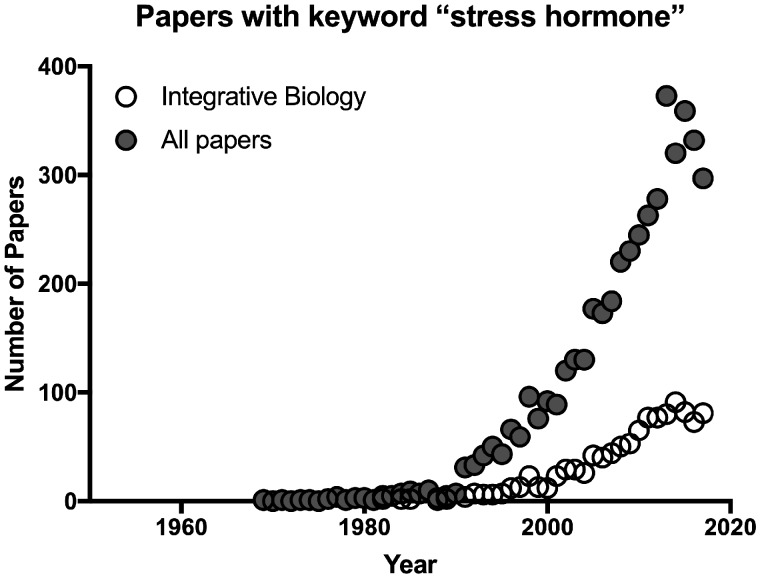
Number of publications per year listed in Web of Science (Clarivate) core collection 1900–2018 that contain the term “stress hormone(s)” in the title or abstract for all journals in the database, and for those journals focusing on Integrative and Comparative Biology.

In recent decades the term “stress hormone” has grown in prevalence in the general endocrinology literature, including in journals focusing on comparative endocrinology and integrative biology (Fig. 1), and is almost exclusively now used in reference to glucocorticoids.

**Fig. 2:**
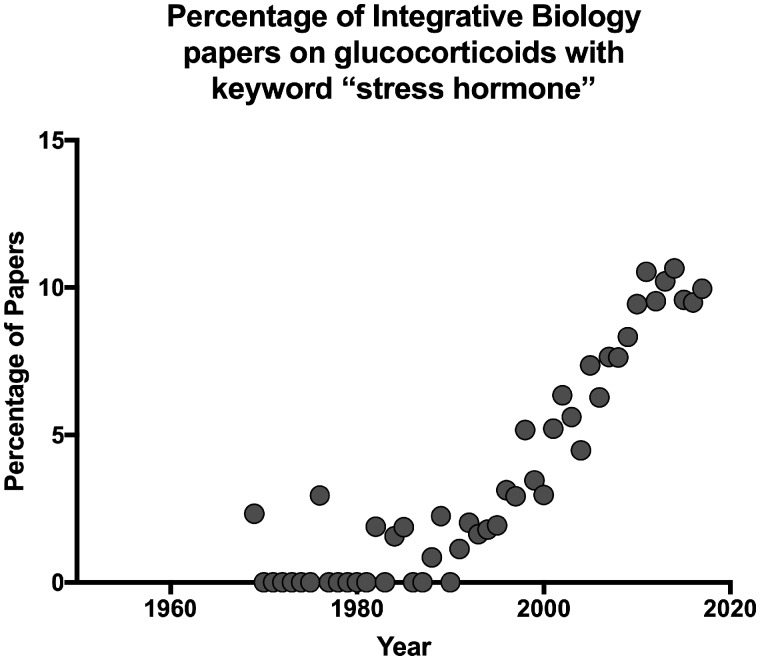
The proportion of publications with a topic of glucocorticoids that contain the phrase “stress hormone(s)” in journals focusing on Integrative and Comparative Biology (listed in Web of Science [Clarivate] core collection 1900–2018). Percentages were calculated by first identifying all papers with the topic of glucocorticoid(s) or cortisol or corticosterone, and then filtering for the phrase stress hormone(s).

Clearly the prevalence of the term “stress hormone” has increased, but so have publication rates in general. More telling is that in journals that focus on integrative and comparative biology, the percentage of papers that have glucocorticoids as a topic that also use the phrase “stress hormone(s)” has increased to about 10% (Fig. 2). Vera et al. (2017) report similar results based on an analysis of 80 publications in *Hormones and Behavior* and *General and Comparative Endocrinology*, two prominent journals in the field. It thus appears that referring to glucocorticoids as stress hormones has increased in the endocrinology literature in general, including in comparative and behavioral endocrinology. In the sections below, we review why this increased usage is problematic.

## The primary function of Cort is not as a stress hormone

One of the primary problems of referring to glucocorticoids as stress hormones is that these hormones have myriad effects that are mediated by multiple receptor types, and only some of these effects play a role in an organism’s response to a stressor. Thus, characterizing glucocorticoids as stress hormones potentially obscures and certainly ignores other important functions. Glucocorticoids play a primary function in energy mobilization including regulating carbohydrate metabolism, hence the name glucocorticoid. Glucocorticoids are critical and essential to life; adrenalectomy without hormone replacement leads to death (Darlington et al. 1990). The widespread effects of glucocorticoids quickly led to their use in a variety of therapeutic settings (David et al. 1970). Glucocorticoids thus have numerous pleiotropic effects and influence the expression of thousands of genes.

How can a single hormone have pleiotropic effects? Glucocorticoids act through binding to multiple types of receptors including intracellular glucocorticoid receptors and mineralocorticoid receptors, as well as membrane-bound receptors (Borski 2000). Further, the two intracellular receptors must form dimers before functioning as transcription factors and can form either homodimers or heterodimers (Trapp et al. 1994; Mifsud and Reul 2016) or even dimerize with receptors for other steroids (Chen et al. 1997). These bound receptors then act as transcription factors, and can promote or suppress expression of thousands of genes. Thus, considering glucocorticoids simply as stress hormones oversimplifies their functions. Glucocorticoids are not stress hormones, but metabolic hormones whose signaling functions have been co-opted as part of a diverse and integrated stress response.

It is also worth remembering that at stress-induced levels glucocorticoids do not create stress. In fact, they are more appropriately referred to as part of the body’s coping mechanisms that facilitate a shift in behavior and physiology so as to minimize the effect of stress on the individual (Romero and Wingfield 2015). That is, during a stress response, elevated glucocorticoids facilitate a shift in energy balance to facilitate coping with a stressor. This function does not make them stress hormones. In fact, the shift in energy balance mediated by glucocorticoids is much more complex than generally acknowledged. Glucocorticoids are well-known to increase plasma glucose levels, but they do so primarily by *decreasing* the use of glucose by most cells of the body. They stimulate the production of a protein that removes glucose transporters from cell membranes, so the increase in plasma glucose substantially results from decreased usage, not increased mobilization (Horner et al. 1987). When combined with the data showing that energy balance modulation primarily occurs 30–120 min after the exposure to a stressor (Munck and Koritz 1962), glucocorticoids would be best to be thought of as mediators of the recovery of a stress response in order to prepare the body for subsequent stressors (Sapolsky et al. 2000).

## Cort is only one part of a complex neural and endocrine vertebrate stress response

The stress response involves a multitude of components that range from the molecular (e.g., heat shock proteins) to the organismal (e.g., sympathetic nervous system activation). Activation of the HPA axis and increases in plasma glucocorticoid concentrations are just one component of that response. Comparative and behavioral endocrinologists are often interested in determining animals’ responses to stressors, and measuring glucocorticoids has become a popular way to do so for everything from basic physiological studies to applied conservation. One reason for their popularity among researchers is that assays for glucocorticoids are widely available and do not require much technical expertise or infrastructure to implement. There is also a substantial literature from biomedicine that connects glucocorticoids to human disease that can be used for interpretation of data. Further, as glucocorticoids are ubiquitous across vertebrates as well as in various tissues that can be minimally or non-invasively sampled, including plasma, saliva, feces, and urine, and integumentary tissues such as hair and feathers, they are routinely used as a snapshot of basic physiological function. It is thus no surprise that studies of stress biology have come to rely so heavily on measures of glucocorticoids as biomarkers of stress, or stress indicators (McCormick and Romero 2017). However, it is critical to keep in mind that measuring glucocorticoid levels is not equivalent to measuring stress. The fact that measuring glucocorticoids alone is not sufficient to characterize the vertebrate stress response has been noted previously (e.g., Breuner et al. 2013). Below we expand on this point using examples and analogies.

Although differences in fecal or plasma glucocorticoid levels across populations may reflect habitat-related differences in exposure to stressors, these differences may reflect other factors. Do the populations differ in phenology? Glucocorticoid levels change with reproductive stage, often increasing during stages associated with high energetic demands like lactation or offspring provisioning, so a difference in phenology could result in a difference in glucocorticoid levels if the study does not carefully account for reproductive stage. Concluding that individual or population differences in glucocorticoids reflect differences in exposure to stressors requires corroborating evidence from other measures, such as body condition or heart rate responses, or, more directly, measures of actual ecological exposures to the stressors of interest. In fact, a recent review of over 200 studies that experimentally induced chronic stress found that glucocorticoids increased, decreased, or remained the same in approximately equal proportions, indicating that glucocorticoid concentrations cannot be used to predict if an individual, or a population, is being exposed to chronic stress (Dickens and Romero 2013). Thus, the use of glucocorticoid levels as a biomarker of stress exposure requires validation for each population studied. Additionally, fitness-related measures are required to interpret the impact of variation in exposure to stressors. Unfortunately, such multivariate approaches are rare. Of 39 papers published in *General and Comparative Endocrinology* in 2017 that report an objective of measuring responses to stressors, two-thirds (23) of them measure only glucocorticoid levels. Moreover, some review articles dedicated to measuring stress in wildlife focus exclusively or almost exclusively on measuring glucocorticoids (e.g., Sheriff et al. 2011). Once again, measures of glucocorticoids in wildlife can be a useful tool, but cannot exclusively be used as biomarkers of exposure to stressors.

The problem with measuring only glucocorticoids when we want to measure exposure to stressors can be illustrated with an analogy. We know that exercise results in increased heart rate. However, just because an animal has an increased heart rate, does not mean that it has been exercising. Heart rate is also elevated during the active portion of the day and during other processes such as digestion. And individuals’ heart rates differ for reasons other than their current exercise state (e.g., due to age, health, etc.). As another example, if you are fighting an infection, your body temperature increases. But an elevated body temperature is not, on its own, convincing evidence of an infection, as body temperature also increases during exercise. These simple examples illustrate the multivariate, complex nature of physiological responses to an array of challenges. In designing our studies, if we focus on the question that motivated the research, we should be able to employ appropriate measures. If we are interested in determining the relative condition or health of an individual or population, direct estimates of fitness might provide more accurate and relevant information than any single physiological measure can offer. If we are interested in understanding physiology, then integrative, multivariate approaches will be most robust.

Also, it is worth considering how one interprets a lack of an increase in glucocorticoid levels. Does this mean that the individual is not experiencing a stressful condition or is it possible it is just activating another mechanism to respond? For example, during molt (the changing of feathers and other integumentary tissues) and during parental care, birds in some populations reduce or even entirely suppress their glucocorticoid response to acute stressors because elevated levels of the hormone could interfere with feather growth or parental care (e.g., Lynn et al. 2003; Walker et al. 2015; Krause et al. 2018). As such, the same stimulus could elicit dramatically different physiological responses based simply on when the animal experiences it. Does this mean that the same event is more stressful during one life history stage than another, or, more likely, that the animal is using different mechanisms to respond most adaptively to the challenge?

## Cort manipulation does not equal stress manipulation

Due to technological and methodological advances, it has become popular in stress biology to manipulate glucocorticoid levels (e.g., Beck et al. 2016) and measure their behavioral, physiological, and life history effects. We have made many significant advances using these techniques. However, a problem has emerged where investigators are manipulating hormone levels and presenting the studies as manipulations of stress. As noted above, glucocorticoids are only one component of a stress response and, further, glucocorticoids have many other roles outside of the organismal stress response.

We present this conflation of manipulating glucocorticoids with manipulating stress as a real problem for our science. First, manipulating glucocorticoid levels leads to negative feedback mechanisms, fundamental changes in the number and distribution of glucocorticoid receptors, and increased clearance. Furthermore, increasing glucocorticoid levels long term is technically challenging and low in ecological validity (Newman et al. 2010; Sopinka et al. 2015; Torres-Medina et al. 2018). Second, and more importantly, we may make false conclusions on how animals respond to stress when glucocorticoid levels are the only independent variable that is manipulated, because other components of the stress response were not. By manipulating only glucocorticoids we cannot disambiguate whether effects on our dependent variables are changes that would occur during a response to a stressor or are due to the multitude of pleiotropic effects of glucocorticoids potentially unrelated to stress responses. There is clear value in manipulating glucocorticoids or the HPA axis to aid in the understanding of whether or not responses to stress are glucocorticoid-dependent. However, such manipulations should be compared directly to other manipulations (e.g., food manipulation; Lendvai et al. 2014). Ideally, a variety of stressors can be manipulated (Cyr et al. 2007) if the goal of the research is to understand the effects of stress and not simply the effects of glucocorticoids.

Is this really a problem, or are we making a straw-man argument? Unfortunately, a substantial number of recent publications that purport to manipulate stress manipulate only glucocorticoids. Of the 23 papers published in *General and Comparative Endocrinology* in 2017 that purport to manipulate stressors, 10 of them only contained manipulations of the HPA axis. This was most common in studies of birds, and less common in studies of fish where manipulations of temperature and oxygen content of water were prevalent. More problematic is the practice of labeling treatment groups as “stressed” when in fact they should be labeled “Cort-treated.” This practice is relatively rare in the published literature, but regularly observed at conference presentations.

## Conclusions and recommendations

Our commentary is not meant to be pedantic, or to target particular researchers or research groups. Rather, we provide these views in the hope they will improve scientific rigor and clarify our understanding of how glucocorticoids play a role in animals’ responses to stressors. The questionable research practice of treating stress and glucocorticoids as synonymous not only obscures our understanding of past research, but also jeopardizes future research.

Here we make some recommendations that we think will help move our field forward.

1. When attempting to measure the response of animals to ecological, social, and environmental stressors, measure more than glucocorticoids. If only measures of glucocorticoids are logistically feasible, acknowledge the limitation of quantifying only one component of a complex response.
2. When attempting to manipulate stress, use multiple stressors. Unless the research question is specific to whether or not glucocorticoids are involved in a response to stressors, manipulating only the HPA axis will not allow for tests of effects of stress, but only tests of effects of a manipulated component of the HPA axis.
3. Use precise language when describing measures and manipulations. Do not equate stress with glucocorticoids, or refer to glucocorticoids as “stress hormones.” Politely correct others who make these mistakes in your roles as reviewers, editors, and mentors.
4. Be clear about what question is being addressed, and the appropriateness/limitations/assumptions of the methods employed to answer the question. In most studies of the ecophysiology of stress, the research question is broader than an exploration of the role of glucocorticoids in a process. Be clear in what the actual question is, and acknowledge that measuring or manipulating glucocorticoids is only one part of addressing the question.
5. Finally, and more generally, scientists should strive to communicate their work to the public, and use plain language whenever possible. However, if the use of plain language obscures and mischaracterizes biological processes, it is counter-productive. Stress is a hot topic in human and animal biology and conflating stress and glucocorticoids will not help the public appreciate the importance of our discoveries. Behavioral endocrinologists should not use phrases such as “stress hormone” any more than they should use the term “male hormone” to refer to testosterone when communicating to journalists or the public. We need to work to communicate simply and clearly, but without sacrificing accuracy. As researchers it is our duty to make sure that the journalists we speak to understand why glucocorticoids and stress are not synonymous. Using phrases such as “stress indicator,” “stress biomarker,” or “stress-associated hormone” would be preferable, but only when describing studies for which glucocorticoids have been validated as a measure of response to stressors.

The success of the recommendations above will depend on the goodwill of our research community to implement them. In addition, we hope that journal editors will include these recommendations in their editorial policies and decision making. As individual researchers, we can work together to address the issues highlighted in this commentary in the collegial spirit that makes our research community productive and rewarding.
